# Supplementary material for: Novel Insights into Milk Coffee Products: Component Interactions, Innovative Processing, and Healthier Product Features
Source: Foods. 2025 Nov 25;14(23):4043. doi: 10.3390/foods14234043 (PMC12691986; doi:10.3390/foods14234043)
Supplement: Supplementary file 1 [file foods-14-04043-s001.zip › foods-3951196-supplementary.pdf]

foods

Table S1. Effect of adding milk to coffee on the antioxidant activity of polyphenols.

Interaction conditions	React	Analyses carried out	Rise/Fall	Reference
Add milk to coffee.	—	HPLC plus diode array detector.	Decreased antioxidant activity of polyphenols.	[1]
Add skim/whole milk to coffee.	Protein-polyphenol interactions.	*In vitro* simulated digestion experiment.	No significant effect.	[2]
Add milk to coffee.	Protein-polyphenol interactions.	*In vitro* simulated digestion experiment.	No significant effect.	[3]
Add whole or skim milk to coffee.	Hydrogen bonding and hydrophobic interactions.	*In vitro* simulated digestion experiment.	No significant effect.	[4]
Add milk to coffee.	Protein-polyphenol interactions.	High performance liquid chromatography, radical cation decolorization method.	Decreased polyphenol content and antioxidant capacity.	[5]
Add milk to coffee.	Protein-polyphenol interactions.	UV-visible spectrophotometry.	Increased antioxidant activity of polyphenols.	[6]
Add milk to coffee.	Complexation with polyphenols in milk via hydrophobic or hydrophilic	—	Increased antioxidant activity of polyphenols.	[7]

	interactions.			
Add full-fat/semi-skimmed/skimmed milk to coffee.	Antioxidants in milk prevent lipid peroxidation and the generation of peroxide/superoxide free radicals.	—	Increased antioxidant activity of polyphenols.	[8]
Add full-fat/semi-skimmed/skimmed milk to coffee.	Hydrophobic lipids interact with the hydrophobic portion of proteins.	H_2O_2 and DPPH method determination.	Increased antioxidant activity of polyphenols.	[9]
Milk added to decaffeinated coffee.	Hydrophobic/or hydrophilic interactions between phenolic acids and milk components.	*In vitro* simulated digestion experiment, high performance liquid phase coupling method.	Increased antioxidant activity of polyphenols.	[10]

Table S2. Effect of milk in coffee on polyphenol bioaccessibility (in vitro).

Interaction conditions	React	Analyses carried out	Increase/Decrease	Reference
Add milk to coffee.	—	HPLC plus diode array detector.	Decreased bioavailability of chlorogenic acid.	[1]
Add milk to coffee.	—	High-performance liquid chromatography, *in vitro* digestion experiments.	Decreased bioavailability of chlorogenic acid.	[11]
Add milk, sugar, or sweetener to coffee.	Protein-phenolic noncovalent interactions.	*In vitro* simulated digestion experiment.	Increased total bioavailability of flavonoids.	[12]
Add skim milk to coffee.	Protein-milk fat interactions.	*In vitro* simulated digestion experiment.	Reduced bioavailability of coffee polyphenols.	[2]
Add skim milk to coffee.	Protein-phenolic noncovalent interactions.	*In vitro* simulated digestion experiment.	Increased bioavailability of coffee polyphenols.	[13]
Add milk to coffee.	Milk micellizes chlorogenic acid.	*In vitro* simulated digestion experiment.	Improved bioaccessibility of chlorogenic acid.	[14]

Adding milk to decaffeinated coffee.	Hydrophobic/or hydrophilic interactions between phenolic acids and milk components.	*In vitro* simulated digestion experiment.	Improved bioavailability of caffeic acid and caffeine.	[10]

Table S3. Comparative assessment of processing routes for milk-coffee beverages.

Method	Scale readiness	Cost / energy	Product impact	Regulatory maturity & validation focus	References
UHT(Ultra-high temperature sterilization)	Fully industrialized; global equipment & supply chain mature	Medium – high thermal energy; CAPEX/OPEX predictable	Achieves commercial sterility; higher thermal load can reduce key volatiles and induce slight "cooked/caramelized" notes	Most mature pathway; validation focuses on commercial sterility (F_0 /cold-spot) and aseptic filling	[15,16]
BAS (blending-after-sterilization)	Medium – high; requires aseptic blending modules added to UHT lines	CAPEX ↑ for aseptic mix manifolds/valving; similar or lower net heat load	Improved aroma retention; mitigates heat-driven flavor drift; mouthfeel/milky notes better preserved	Clear regulatory route but stricter aseptic post-UHT blending environmental/monitoring validation	[17]
HPH (high-pressure homogenization)	Medium (widely used in dairy for quality & shelf-life support)	Electrical energy medium – high; higher pressure ↑ wear/energy	Smaller droplet size/less creaming, finer interfaces; can support antioxidant retention and sensory stability	Equipment/validation mature; watch mechanical wear/temperature rise and shelf-life substantiation	[18]
PEF (pulsed electric fields)	Low – medium; commercial in juices, accelerating in dairy	Higher CAPEX; specific energy medium	Near-ambient pasteurization/sterilization equivalents; better nutrient/volatile preservation vs. high heat	Growing maturity; needs equivalent lethality, equipment safety, and shelf-life evidence	[19]
CP (cold plasma)	Lab – pilot; continuous dairy lines emerging	Low thermal load; electrical use depends on discharge	Low-temperature decontamination with good color/volatile/vitamin retention; over-treatment risks reactive-species off-notes or packaging compatibility	Developing; focus on reactive species residues/by-products, packaging interactions, safety	[20]
Ultrasound (power ultrasound)	Pilot – industrial as auxiliary unit (often coupled with homogenization/heat)	Electrical/mechanical energy medium; scale-up needs power density control	Promotes dispersion and droplet size reduction; aids extraction/interface remodeling; overuse may trigger oxidation/off-flavor	Validate temperature rise control, quality & safety endpoints; typically adjunct step	[21]

[1] Jeon, J.; Kim, H.; Jeong, I.; Hong, S.; Oh, M.; Yoon, M.; Shim, J.; Jeong, J. H.; & El-Aty, A. M. A.. Contents of chlorogenic acids and caffeine in various coffee-related products. *Journal of Advanced Research (Online)***2019**, 17, 85–94. https://doi.org/10.1016/j.jare.2019.01.002

[2] Quan, W.; Qie, X.; Chen, Y.; Zeng, M.; Qin, F.; Chen, J.; & He, Z.. Effect of milk addition and processing on the antioxidant capacity and phenolic bioaccessibility of coffee by using an in vitro gastrointestinal digestion model. *Food Chemistry***2020**, 308, 125598. https://doi.org/10.1016/j.foodchem.2019.125598

[3] Otemuyiwa, I. O.; Williams, M. F.; & Adewusi, S. A.. Antioxidant activity of health tea infusions and effect of sugar and milk on in-vitro availability of phenolics in tea, coffee and cocoa drinks. *Nutrition & Food Science***2017**, 47(4), 458–468. https://doi.org/10.1108/nfs-08-2016-0134

[4] Davide, T.; Ahmed, H.; Elena, V.; & Angela, C.. The Type and Concentration of Milk Increase the in Vitro Bioaccessibility of Coffee Chlorogenic Acids. *Journal of Agricultural and Food Chemistry***2012**. https://doi.org/10.1021/jf302694a

[5] Niseteo, T.; Komes, D.; Belščak-Cvitanović, A.; Horžić, D.; & Budeč, M.. Bioactive composition and antioxidant potential of different commonly consumed coffee brews affected by their preparation technique and milk addition. *Food Chemistry***2012**, 134(4), 1870–1877. https://doi.org/10.1016/j.foodchem.2012.03.095

[6] Komes, D.; Bušić, A.; Vojvodić, A.; Belščak-Cvitanović, A.; & Hruškar, M.. Antioxidative potential of different coffee substitute brews affected by milk addition. *European Food Research & Technology***2015**, 241(1), 115–125. https://doi.org/10.1007/s00217-015-2440-z

[7] Bandyopadhyay, P.; Ghosh, A.; & Ghosh, C.. Recent developments on polyphenol–protein interactions: effects on tea and coffee taste, antioxidant properties and the digestive system. *Food & Function (Print)***2012**, 3(6), 592. https://doi.org/10.1039/c2fo00006g

[8] Ryan, L.; & Petit, S.. Addition of whole, semiskimmed, and skimmed bovine milk reduces the total antioxidant capacity of black tea. *Nutrition Research***2010**, 30(1), 14–20. https://doi.org/10.1016/j.nutres.2009.11.005

[9] Al-Ghafari, A. B.; Alharbi, R.; Al-Jehani, M. M.; Bujeir, S. A.; Doghaither, H. a. A.; & Omar, U. M.. The effect of adding different concentrations of cows' milk on the antioxidant properties of coffee. *Biosciences, Biotechnology Research Asia***2017**, 14(1), 177–184. https://doi.org/10.13005/bbra/2433

[10] Soares, M. J.; De Souza Figueira, M.; Sampaio, G. R.; Soares-Freitas, R. a. M.; Da C Pinaffi-Langley, A. C.; & Da Silva Torres, E. a. F.. Coffee simulated inhibition of pancreatic lipase and antioxidant activities: Effect of milk and decaffeination. *Food Research International***2022**, 160, 111730. https://doi.org/10.1016/j.foodres.2022.111730

[11] Duarte, G.; & Farah, A.. Effect of simultaneous consumption of milk and coffee on chlorogenic acids' bioavailability in humans. *Journal of Agricultural and Food Chemistry***2011**, 59(14), 7925–7931. https://doi.org/10.1021/jf201906p

[12] Kamiloglu, S.; Özdal, T.; Bakır, S.; & Çapanoğlu, E.. Bioaccessibility of terebinth (Pistacia terebinthus L.) coffee polyphenols: Influence of milk, sugar and sweetener addition. *Food Chemistry***2022**, 374, 131728. https://doi.org/10.1016/j.foodchem.2021.131728

[13] Qie, X.; Cheng, Y. M.; Chen, Y.; Zeng, M.; Wang, Z.; Qin, F.; Chen, J.; Li, W.; & He, Z. . In vitro phenolic bioaccessibility of coffee beverages with milk and soy subjected to thermal treatment and protein–phenolic interactions. *Food Chemistry***2022**, 375, 131644. https://doi.org/10.1016/j.foodchem.2021.131644

[14] Alongi, M.; Calligaris, S.; & Anese, M.. Fat concentration and high-pressure homogenization affect chlorogenic acid bioaccessibility and α-glucosidase inhibitory capacity of milk-based coffee beverages.*Journal of Functional Foods***2019**, 58, 130-137. https://doi.org/10.1016/j.jff.2019.04.057

[15] Deeth, H. C., & Lewis, M. J. (2017). High temperature processing of milk and milk products. John Wiley & Sons.

[16] Burton, H. (2012). Ultra-high-temperature processing of milk and milk products. Springer Science & Business Media.

[17] Ikeda, M.; Akiyama, M.; Hirano, Y.; Miyazi, K.; Kono, M.; Imayoshi, Y.; Iwabuchi, H.; Onodera, T.; & Toko, K.. Effects of Processing Conditions During Manufacture on Retronasal-Aroma Compounds from a Milk Coffee Drink. *Journal of Food Science* **2018a**, 83(3), 605–616. https://doi.org/10.1111/1750-3841.14054

[18] Pereda, J., Ferragut, V., Quevedo, J. M., Guamis, B., & Trujillo, A. J. (2007). Effects of ultra-high pressure homogenization on microbial and physicochemical shelf life of milk. Journal of dairy science, 90(3), 1081-1093.

[19] Buckow, R., Chandry, P. S., Ng, S. Y., McAuley, C. M., & Swanson, B. G. (2014). Opportunities and challenges in pulsed electric field processing of dairy products. International Dairy Journal, 34(2), 199-212.

[20] Misra, N. N., Patil, S., Moiseev, T., Bourke, P., Mosnier, J. P., Keener, K. M., & Cullen, P. J. (2014). In-package atmospheric pressure cold plasma treatment of strawberries. Journal of food engineering, 125, 131-138.

[21] Chemat, F., Rombaut, N., Sicaire, A. G., Meullemiestre, A., Fabiano-Tixier, A. S., & Abert-Vian, M. (2017). Ultrasound assisted extraction of food and natural products. Mechanisms, techniques, combinations, protocols and applications. A review. Ultrasonics sonochemistry, 34, 540-560.
